# Supplementary material for: Characterizing trachoma elimination using serology
Source: Nat Commun. 2025 Jul 1;16:5545. doi: 10.1038/s41467-025-60581-z (PMC12216694; doi:10.1038/s41467-025-60581-z)
Supplement: Supplementary file 1 — Supplementary Information [file 41467_2025_60581_MOESM1_ESM.pdf]

SUPPLEMENTARY INFORMATION

Supplementary Information Text

Clinical assessment and serological testing

Clinical examination was undertaken by trained field graders using the WHO simplified grading system [1]. Conjunctival swabs were passed over the conjunctiva three times, rotating 120° between passes, and tested for *C. trachomatis* DNA using PCR either as individual or pooled samples, as previously described [2]. Blood spots for serology assays were collected onto filter papers and most often tested in a multiplex bead assay for IgG antibodies to the Pgp3 and CT694 antigens, as previously described [3]. For this, antigen-coupled beads were mixed with control sera and blood spot eluates and total IgG determined by reading the beads on a Luminex instrument (Luminex Corp., Austin, TX) and converting the signal readout to median fluorescence intensity (MFI) with background levels subtracted out (MFI-BG). Seropositivity thresholds were generated using receiver-operating characteristic curves of specimens of previously classified positive or negative samples [4]. Other established platforms for detection of anti-Pgp3 antibody, ELISA and lateral flow assays (LFA), were also used in various contexts, as previously described [5–9]. The different serology testing platforms are generally closely comparable [4]. Thresholds to determine sero-positivity were defined for each study or dataset separately.

Further details of study design, clinical examination, sampling methodology and laboratory procedures can be found in publications for the respective individual studies (Table S1).

Estimation of seroconversion rate within a generalized linear model using maximum likelihood

The seroconversion rate (SCR) from a current status, single-rate catalytic model assuming no seroreversion is closely tied to the slope of age-seroprevalence curve. The SCR is equal to the slope of the age-seroprevalence curve divided by the complement of the seroprevalence at age A = a. It can be shown that the SCR based on this model can be estimated as the exponentiated intercept from a generalized linear model with binomial error structure and a complementary log-log link:

$$log[-\log(1-P(Y=1|A))] = log\lambda + logA$$

where Y represents individual-level serostatus (1: seropositive, 0: seronegative or equivocal), A is the child's age in years, and λ is the SCR. The SCR provides an estimate of the force of

infection, an epidemiological parameter that denotes the rate at which susceptible individuals in the population become infected.

Reversible catalytic model

Next, we extended the model above to allow for seroreversion. Cross-sectional data do not contain sufficient information to reliably estimate both seroreversion and seroconversion rates. We fit a binomial maximum likelihood model for seroconversion with fixed a seroreversion rate of 6.6 per 100 person-years based on a longitudinal cohort in Kongwa, United Republic of Tanzania, monitored in the absence of MDA [10]:

$$P(Y = 1|A) = \frac{\lambda}{\lambda + \rho}[1 - e^{-(\lambda+\rho)(A)}]$$

where ρ is the assumed seroreversion rate.

As an internal validity check, we compared the SCR estimates from the simple and reversible models.

Alternative categories based on elimination of transmission

The primary analysis categorized EUs based on their need for population-level trachoma-specific interventions. In many cases, populations could reach a level of trachoma control that would not justify further large-scale interventions, such as antibiotic mass drug administration (MDA), yet still have underlying detectable ocular *C. trachomatis* transmission. We conducted an alternative analysis that categorized EUs by level of *C. trachomatis* transmission defined using clinical data (prevalence of trachomatous inflammation—follicular, TF), prevalence of infection by PCR, and programmatic context such as recent MDA application, with a goal of identifying a subgroup of EUs that had likely achieved interruption of transmission. A caveat of the analysis is that there are no formal benchmarks or international guidance for documenting elimination of ocular *C. trachomatis* transmission, but we report these results as an illustrative example for how the problem could be approached. It also provides an example of how the methodology used can generalize to include multiple transmission categories (not only two as in the primary analysis).

We created three categories: (i) `Probable interruption of transmission` – (n=14) – which comprised EUs with population-level TF of <5% in 1-9-year-olds, ocular infection prevalence of <1%, and no recent or planned MDA treatments for a minimum of one year prior to the survey, as MDA can transiently reduce infection prevalence. (ii) `Endemic` – (n=7) – EUs with unambiguously high transmission, as evident by high TF prevalence (>10%), high infection prevalence (>5%) and where MDA programs were in place or additional MDA treatments were

planned. (iii) `Near interruption of transmission` – (n=14) – comprising EUs with intermediate clinical and PCR measures that were not at either extreme described above. The near interruption of transmission EUs were thought as being on the path to interruption of transmission but with unusual epidemiology (e.g., higher prevalence of clinical signs or infection than would be expected) or requiring an additional round of MDA or monitoring to assess disease status. Finally, EUs with IgG measurements that lacked clinical or PCR measures were used as an independent, held-out (or test-set) sample to assess the methodology (n=13). It should be noted that the infection thresholds used here to demarcate the interruption of transmission and endemic categories are strictly hypothetical and should not be interpreted as factual, or as representing the views, decisions or policies of any organization contributing to the fight for global trachoma elimination.

We used the same approach as the primary analysis to make probability statements for each transmission category, a 3-component mixture model for the pooled distributions of SCR estimates. This approach assumed that each MCMC estimate is drawn independently from a multinomial distribution made of three components parallel to the three transmission categories defined above, $k \in \{1,2,3\}$. So, for each transmission category (C_k: probable interruption of transmission, near interruption of transmission, endemic) and SCR estimate, $x \epsilon \mathbb{R}$, we compute the posterior probability, $p(C_k|x)$, using the Bayes rule:

$$p(C_k|x) \propto p(C_k) * \frac{p(x|C_k)}{p(x)}$$

where $p(C_k)$ is the prior probability that a population is in transmission category C_k; and $p(x|C)$, is the likelihood evaluated as empirical probability density function at each MCMC draw x. $p(x)$ denotes the marginal likelihood or normalizing constant for the posterior density obtained by integrating the products of the likelihood, $p(x|C_k)$, and the prior probability. That is, the sum of the products of the density function and prior probability for each k,

$$\sum_{k=1}^{3} \omega_k f_k(x|C_k)$$

$p(x|C)/p(x)$ forms the likelihood ratio in the Bayesian mixture model. The prior probabilities were defined such that they sum up to one, i.e., $\sum_k \omega_k = 1$. We used a general quadratic function $f(x) = ax^2 + bx + c$ such as,

$$a = b = p\left(C_{k=Probable\ interruption\ of\ transmission}\right)$$

$$c = p\left(C_{k=Probable\ interruption\ of\ transmission}\right) - 1$$

The positive root, x, of the quadratic equation above was used to determine the prior probabilities of the near interruption of transmission and endemic categories as,

$$p\left(C_{k=Near\ interruption\ of\ transmission}\right) = a * x$$

$$p(C_{k=Endemic}) = a * x^2$$

Of the 48 EUs, 35 had information on TF and infection prevalence and could be classified into the three transmission categories defined above. The remaining unclassified EUs in this supplementary analysis (n=13) were treated as unclassified set of surveys and used with the mixture model approach to estimate their posterior probability of each transmission category.

SCR estimates were generally well separated between trachoma transmission categories defined using clinical and infection measures. SCRs were low among the EUs in the probable interruption of transmission category, with the median SCR in the age group 1-5 years estimated between 0.1 (95% CrI, 0.0 to 0.2) and 0.9 (0.5 to 1.6) per 100 person-years. In the near interruption of transmission category, the estimated SCRs ranged from 0.5 (0.2-1) to 4.1 (3.1-5.5) per 100 person-years. There was substantial heterogeneity in SCR estimates among EUs in the endemic transmission category, with median estimates as high as 17.6 (13.6-22.3) per 100 person-years.

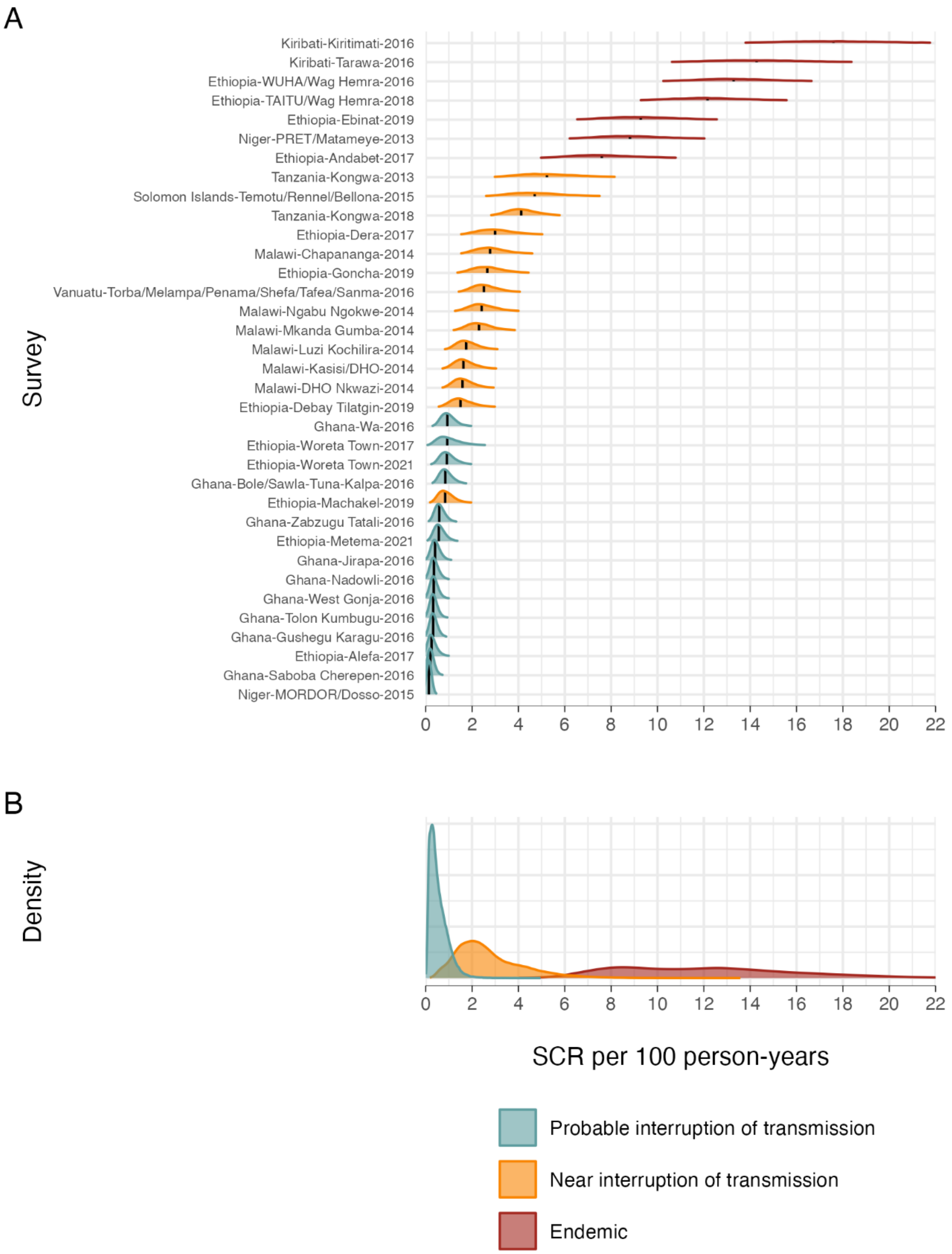
A
Kiribati-Kiritimati-2016
Kiribati-Tarawa-2016
Ethiopia-WUHA/Wag Hemra-2016
Ethiopia-TAITU/Wag Hemra-2018
Ethiopia-Ebinat-2019
Niger-PRET/Matameye-2013
Ethiopia-Andabet-2017
Tanzania-Kongwa-2013
Solomon Islands-Temotu/Rennel/Bellona-2015
Tanzania-Kongwa-2018
Ethiopia-Dera-2017
Malawi-Chapananga-2014
Ethiopia-Goncha-2019
Vanuatu-Torba/Melampa/Penama/Shefa/Tafea/Sanma-2016
Malawi-Ngabu Ngokwe-2014
Malawi-Mkanda Gumba-2014
Malawi-Luzi Kochilira-2014
Malawi-Kasisi/DHO-2014
Malawi-DHO Nkwazi-2014
Ethiopia-Debay Tilatgin-2019
Ghana-Wa-2016
Ethiopia-Woreta Town-2017
Ethiopia-Woreta Town-2021
Ghana-Bole/Sawla-Tuna-Kalpa-2016
Ethiopia-Machakel-2019
Ghana-Zabzugu Tatali-2016
Ethiopia-Metema-2021
Ghana-Jirapa-2016
Ghana-Nadowli-2016
Ghana-West Gonja-2016
Ghana-Tolon Kumbugu-2016
Ghana-Gushegu Karagu-2016
Ethiopia-Alefa-2017
Ghana-Saboba Cherepen-2016
Niger-MORDOR/Dosso-2015
Survey
0 2 4 6 8 10 12 14 16 18 20 22
B
Density
0 2 4 6 8 10 12 14 16 18 20 22
SCR per 100 person-years
Probable interruption of transmission
Near interruption of transmission
Endemic

With a moderately informative prior of 80% probability of elimination, the posterior probability that an EU is in the post elimination category exceeds 90% when the SCR is = 1.0 per 100 person-years.

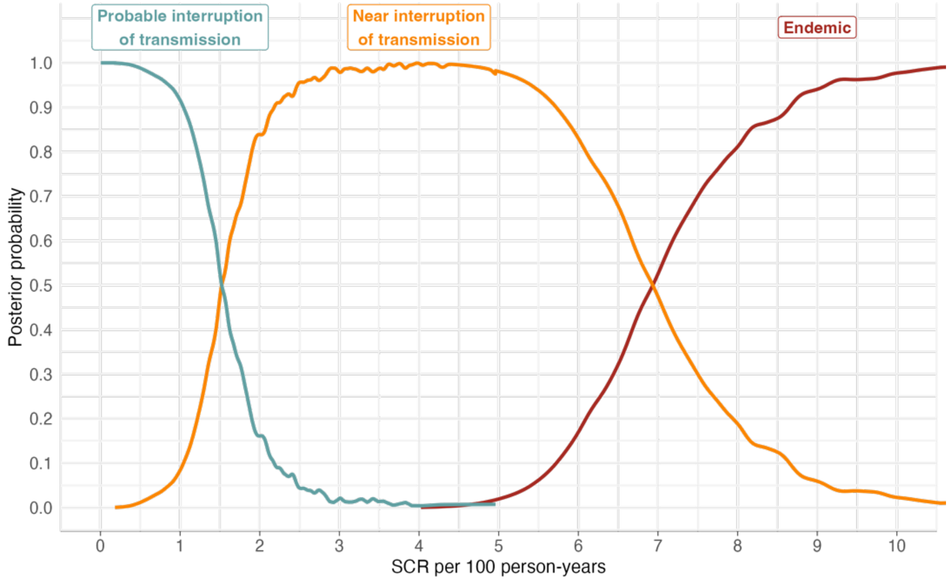

For each unclassified EU, we calculated the posterior probability of its membership in each transmission category given its SCR distribution, assuming informative prior probabilities of 80% elimination, 17% near elimination, and 3% endemic categories.

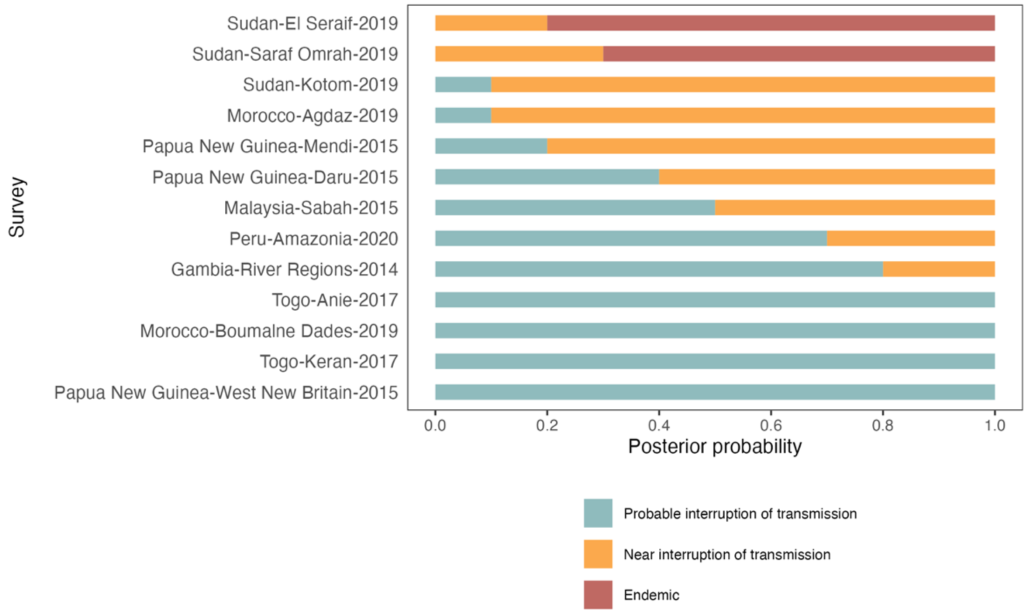

Supplementary Information Tables

Supplementary Table 1. Evaluation unit-level seroconversion rates (SCR) per 100 person-years among children 1–5-years-old corresponding with different trachoma programmatic decisions (Action not needed, Action needed) across varying prior probability assumptions and levels of certainty based on the posterior probability of each category.

Prior Probability	Level of Certainty	SCR threshold Action not needed	SCR threshold Action needed
80% Action not needed / 20% Action needed	99%	1.3	7.4
	95%	1.8	5.1
	90%	2.2	4.5
	80%	2.8	4.1
50% Action not needed / 50% Action needed	99%	1.0	7.4
	95%	1.4	4.2
	90%	1.6	3.8
	80%	1.9	3.4
20% Action not needed / 80% Action needed	99%	*	7.4
	95%	1.0	3.6
	90%	1.2	3.2
	80%	1.4	2.8

* There was no SCR threshold value for the `Action not needed` programmatic decision corresponding with 99% certainty under a weak prior probability of 20% Action not needed.

Supplementary Table 2. Comparison of expert reviewers' classification of evaluation unit (EU) category with the predicted probability of being in a category in a leave-one-out (LOO) sensitivity analysis. Ten reviewers were asked to classify EUs into three categories, and in the case of a consensus classification (≥70%) reviewer classifications were used as class labels in the primary analysis. A jackknife *n-1* leave out (LOO) sensitivity analysis repeated the model fit iteratively, in each iteration one of the 34 EUs was left out in turn (see Methods). For each left-out EU, its predicted probability of Action not needed / Action needed was obtained from the model fit on data from the remaining 33 EUs and is summarized in this table. Note that only 34/48 EUs were classified in the Action not needed and Action needed categories by the expert reviewers since these had complete information on TF, PCR and serology measurements, or had achieved the Elimination of Public Health Problem (EPHP) status (The Gambia, Togo and Morocco).

	**Expert reviewer votes**				**Predicted probability (%) of category in a LOO analysis**	
**Survey (Country-EU-Year)**	**Action not needed**	**Unclassified**	**Action needed**	**Consensus (≥70%)**	**Action not needed**	**Action needed**
Ethiopia-Alefa-2017	10			Action not needed	100	0
Ethiopia-Andabet-2017			10	Action needed	0	100
Ethiopia-Ebinat-2019			10	Action needed	0	100
Ethiopia-Goncha-2019		3	7	Action needed	75	25
Ethiopia-Metema-2021	10			Action not needed	100	0
Ethiopia-TAITU/Wag Hemra-2018			10	Action needed	0	100
Ethiopia-Woreta Town-2017	10			Action not needed	99	1
Ethiopia-Woreta Town-2021	9	1		Action not needed	100	0
Ethiopia-WUHA/Wag Hemra-2016	1		9	Action needed	0	100
Gambia-River Regions-2014				Action not needed	99	1
Ghana-Bole/Sawla-Tuna-Kalpa-2016	10			Action not needed	100	0
Ghana-Gushegu Karagu-2016	10			Action not needed	100	0
Ghana-Jirapa-2016	10			Action not needed	100	0
Ghana-Nadowli-2016	10			Action not needed	100	0
Ghana-Saboba Cherepen-2016	10			Action not needed	100	0
Ghana-Tolon Kumbugu-2016	10			Action not needed	100	0
Ghana-Wa-2016	10			Action not needed	100	0
Ghana-West Gonja-2016	10			Action not needed	100	0
Ghana-Zabzugu Tatali-2016	10			Action not needed	100	0
Kiribati-Kiritimati-2016			10	Action needed	0	100
Kiribati-Tarawa-2016			10	Action needed	0	100
Malawi-Chapananga-2014	7	3		Action not needed	71	29
Malawi-DHO Nkwazi-2014	7	3		Action not needed	96	4
Malawi-Kasisi/DHO-2014	7	3		Action not needed	95	5
Malawi-Luzi Kochilira-2014	7	3		Action not needed	94	6

Morocco-Agdaz-2019				Action not needed	81	19
Morocco-Boumalne Dades-2019				Action not needed	100	0
Niger-MORDOR/Dosso-2015	10			Action not needed	100	0
Niger-PRET/Matameye-2013	1		9	Action needed	0	100
Solomon Islands-Temotu/Rennel/Bellona-2015		2	8	Action needed	17	84
Tanzania-Kongwa-2013		2	8	Action needed	10	91
Tanzania-Kongwa-2018		3	7	Action needed	23	77
Togo-Anie-2017				Action not needed	100	0
Togo-Keran-2017				Action not needed	100	0

TF = trachomatous inflammation—follicular; PCR = polymerase chain reaction

Supplementary Table 3. Comparison of ten expert reviewer votes for the unclassified evaluation units (EU) with the predicted category probabilities. Expert consensus for category membership of each EU was ≥ 70% of the votes. See Methods and Supplementary Text for definition of the categories. Note that only 9/14 unclassified EUs were available for expert reviewer classification since they had TF, PCR and serology measurements. The comparisons of these nine are shown below. We conducted a three-category supplementary analysis, in which the three categories were Probable interruption of transmission (PIT), Near interruption of transmission (NIT) and Endemic transmission (ET) (see Supplementary Information Text). In the supplementary analysis, only three out of the nine unclassified EUs in the primary analysis were held-out or categorized as unclassified, which explains the gaps in the last three columns in the table below. That is, the primary and supplementary analyses had different definitions and requirements for category membership, and therefore did not have matching sets of EUs in the various categories.

	**Expert reviewer votes for category**			**Predicted category probability (%) for respective category**				
**Survey (Country-EU-Year)**	**Action not needed**	**Uncla-ssified**	**Action Needed**	**Action not needed**	**Action Needed**	**PIT**	**NIT**	**ET**
Papua New Guinea-Daru-2015 *	1	9		94.2	5.8	39	60.9	0.2
Ethiopia-Dera-2017		8	2	63.4	36.6			
Malawi-Mkanda Gumba-2014	6	4		84.9	15.1			
Malawi-Ngabu Ngokwe-2014	6	4		82.1	17.9			
Papua New Guinea-Mendi-2015 *	1	7	2	93.2	6.8	24.8	75.2	0
Papua New Guinea-West New Britain-2015 *	2	8		99.9	0.2	97.9	2.1	0
Vanuatu-Torba/Melampa/Penama/Shefa/Tafea/Sanma-2016	2	5	3	80.3	19.7			
Ethiopia-Debay Tilatgin-2019		5	5	96	4			
Ethiopia-Machakel-2019	3	6	1	99.6	0.4			

TF = trachomatous inflammation—follicular; PCR = polymerase chain reaction

Supplementary Table 4. Characteristics of the studies and sources of datasets analyzed here.

Country	Region / District / Evaluation unit(s)	Year of survey	Serology assay	Relevant publication(s)
Ethiopia	Alefa	2017	MBA	PMID: 33200728
Ethiopia	Andabet	2017	MBA	PMID: 33200728
Ethiopia	Debay Tilatgin	2019	MBA	PMID: 40067808
Ethiopia	Dera	2017	MBA	PMID: 33200728
Ethiopia	Ebinat	2019	MBA	PMID: 40067808
Ethiopia	Goncha	2019	MBA	PMID: 40067808
Ethiopia	Machakel	2019	MBA	PMID: 40067808
Ethiopia	Metema	2021	MBA	PMID: 38386689
Ethiopia	Wag Hemra	2018	MBA	PMID: 32877398
Ethiopia	Wag Hemra	2016	MBA	PMID: 34919861
Ethiopia	Woreta Town	2017	MBA	PMID: 33200728
Ethiopia	Woreta Town	2021	MBA	PMID: 38386689
The Gambia	Lower & Upper River Regions	2014	ELISA	PMID: 28099433
Ghana	Bole; Sawla-Tuna-Kalpa	2016	MBA	PMID: 30550537
Ghana	Gushegu Karagu	2016	MBA	PMID: 30550537
Ghana	Jirapa	2016	MBA	PMID: 30550537
Ghana	Nadowli	2016	MBA	PMID: 30550537
Ghana	Saboba Cherepen	2016	MBA	PMID: 30550537
Ghana	Tolon Kumbugu	2016	MBA	PMID: 30550537
Ghana	Wa	2016	MBA	PMID: 30550537
Ghana	West Gonja	2016	MBA	PMID: 30550537
Ghana	Zabzugu Tatali	2016	MBA	PMID: 30550537
Kiribati	Kiritimati	2016	MBA	PMID: 28898240
Kiribati	Tarawa	2016	MBA	PMID: 28898240
Malawi	Chapananga	2014	ELISA	PMID: 31658258
Malawi	DHO Nkwazi	2014	ELISA	PMID: 31658258
Malawi	Kasisi/DHO	2014	ELISA	PMID: 31658258
Malawi	Luzi Kochilira	2014	ELISA	PMID: 31658258
Malawi	Mkanda Gumba	2014	ELISA	PMID: 31658258
Malawi	Ngabu Ngokwe	2014	ELISA	PMID: 31658258
Malaysia	Sabah	2015	MBA	PMID: 36388287
Morocco	Agdaz	2019	MBA	PMID: 35344929
Morocco	Boumalne Dades	2019	MBA	PMID: 35344929
Niger	Dosso	2015	MBA	PMID: 31237871
Niger	Matameye	2013	MBA	PMID: 27956455

Papua New Guinea	Daru	2015	MBA	PMID: 31965155
Papua New Guinea	Mendi	2015	MBA	PMID: 31965155
Papua New Guinea	West New Britain	2015	MBA	PMID: 31965155
Peru	Amazonia region	2020	MBA	In peer review
Solomon Islands	Temotu; Rennel; Bellona	2015	MBA	PMID: 29588922
Sudan	El Seraif	2019	MBA	PMID: 38507810
Sudan	Kotom	2019	MBA	PMID: 38507810
Sudan	Saraf Omrah	2019	MBA	PMID: 38507810
Tanzania	Kongwa	2013	MBA	PMID: 30543311
Tanzania	Kongwa	2018	MBA	PMID: 33861754
Togo	Anie	2017	MBA	PMID: 33790370
Togo	Keran	2017	MBA	PMID: 33790370
Vanuatu	Torba; Malampa; Penama; Shefa; Tafea; Sanma	2016	MBA	PMID: 32017971

* PMID = PubMed ID. ELISA = enzyme-linked immunosorbent assay. MBA = multiplex bead assay.

Supplementary Information Figures

Supplementary Fig 1. Seroconversion rate (SCR) per 100 person-years in 1–5-year-olds (N=41,168) – using a catalytic model assuming constant force of infection. Density distributions of the SCR for 48 evaluation units (EUs). The black vertical line shows the median estimate, and the density distributions depict the uncertainty about the median. EUs are colored by categories based on programmatic responses (Methods) and ordered by increasing median SCR value.

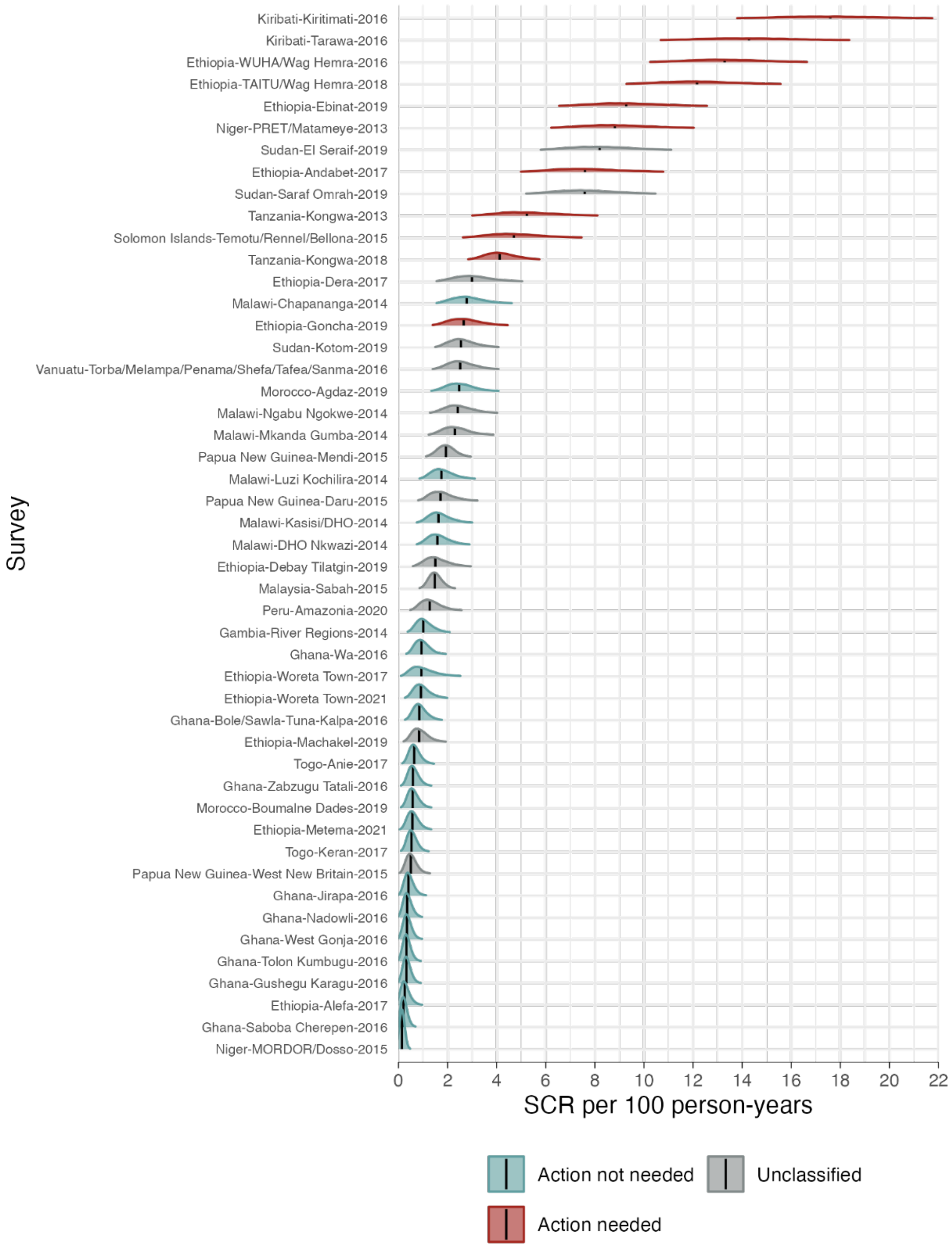

Fig S2. Relationship between the seroconversion rate (SCR) and seroprevalence at the evaluation unit (EU) level among 1–5-year-olds. EUs are colored by transmission categories used in the main analysis (N=48 EUs). The right panel provides a zoomed view of estimates for populations approaching elimination, illustrating a highly linear relationship between serological summary measures. A linear regression fit to the 41 EUs with seroprevalence ≤25% (the linear range of the curve) led to the model with intercept and slope: $SCR \sim 0 + 0.363 \times Seroprev$ (R^2 =0.99), with SCR in units per 100 and seroprevalence in percentage points.

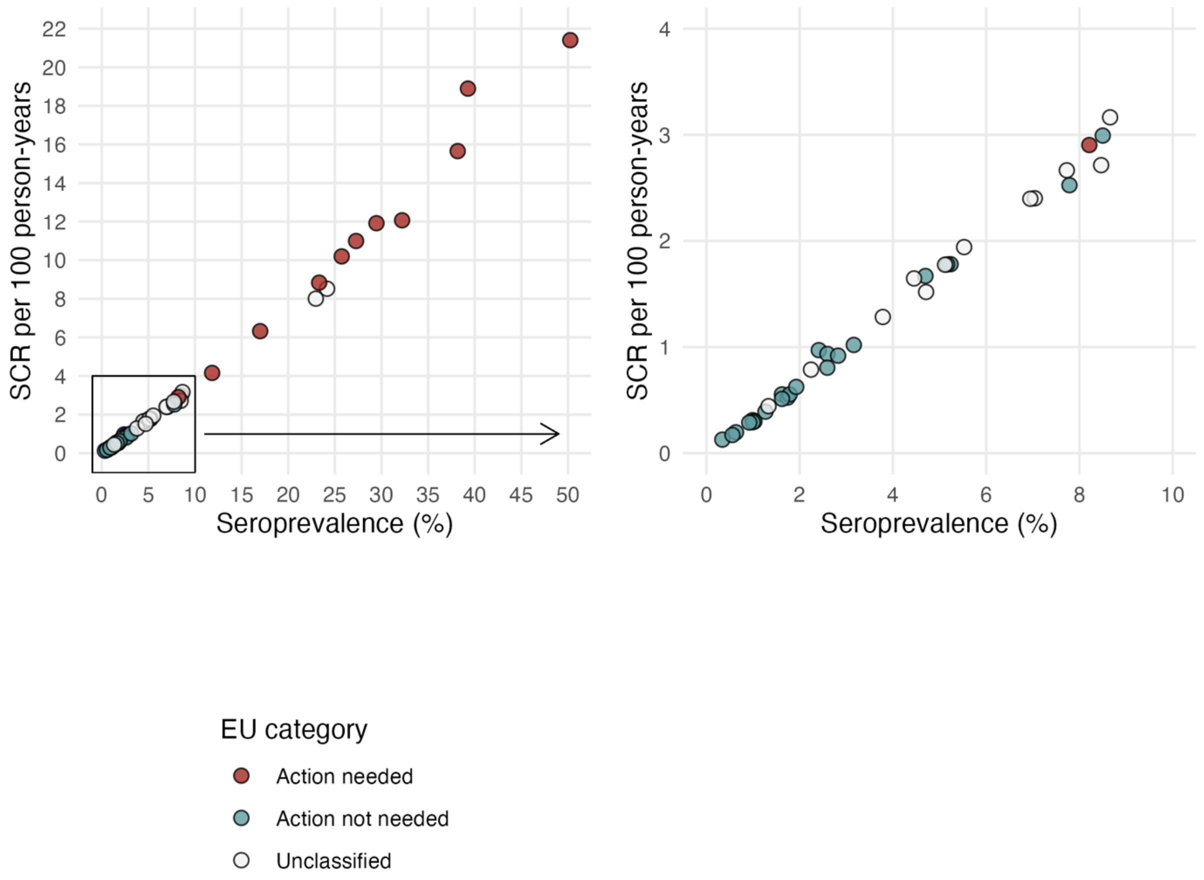

Fig S3. Seroprevalence at evaluation unit (EU) level in 1–5-year-olds. Seroprevalence was estimated by fitting generalized linear mixed models using Bayesian Markov chain Monte Carlo methods (see Methods). **(A)** Posterior density distributions of seroprevalence for each EU. The median estimate is showed by a black vertical line and the density distributions depict the uncertainty about the median. EUs are ordered by increasing median value and colored by programmatic action categories of whether additional trachoma-specific interventions are needed (Methods). **(B)** Pooled density distributions for each category.

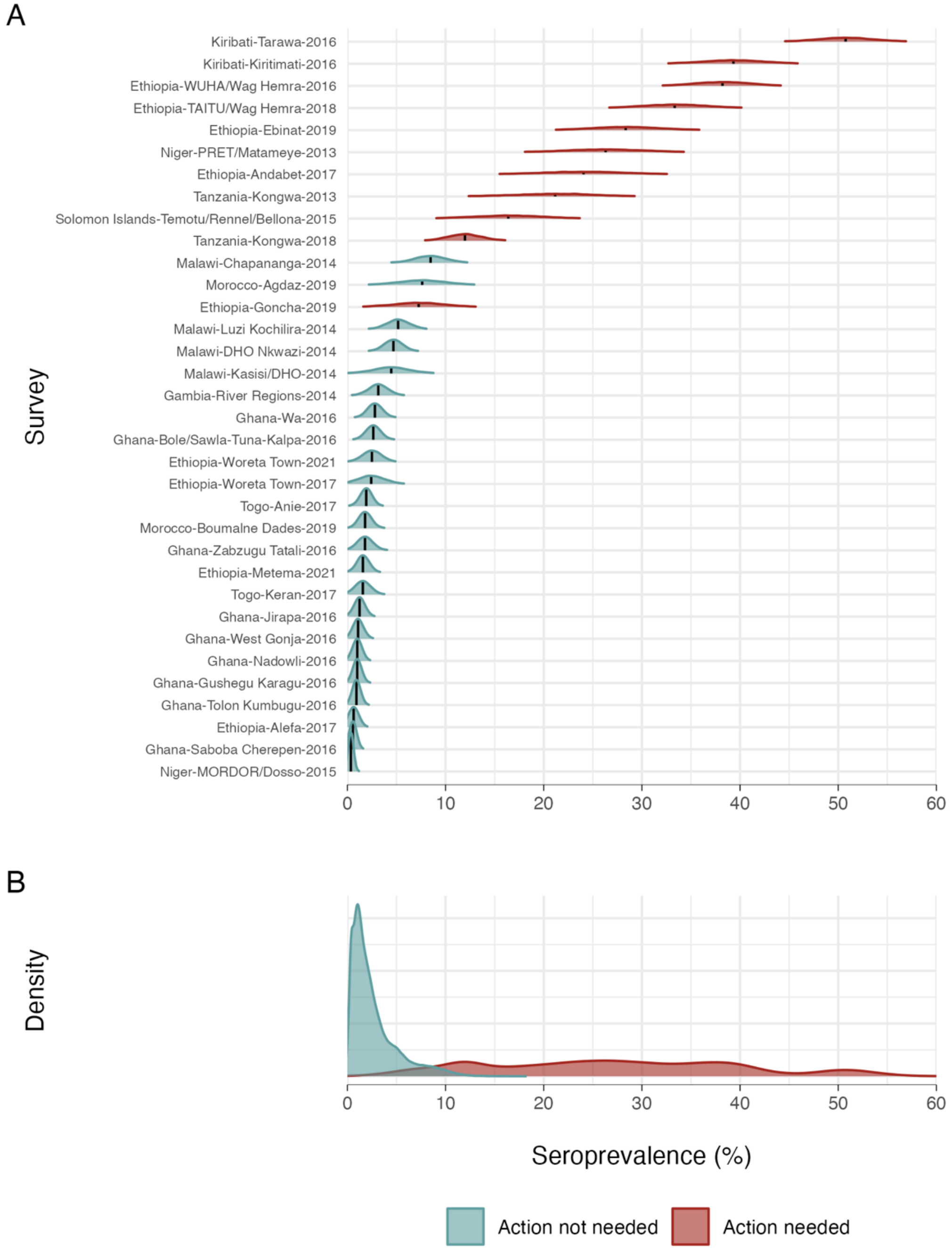

Fig S4. Sensitivity analysis of posterior probability of categories given the seroconversion rate. Evaluation units were grouped into Action not needed and Action needed categories and the pooled SCR estimates used in a Bayesian mixture model to obtain posterior probabilities (Methods). The plots depict posterior probabilities of each category for a range of weakly and moderately informative prior probabilities. We considered stronger priors for the Action not needed category in panel **(A)**, and stronger priors for the Action needed category in panel **(B)**.

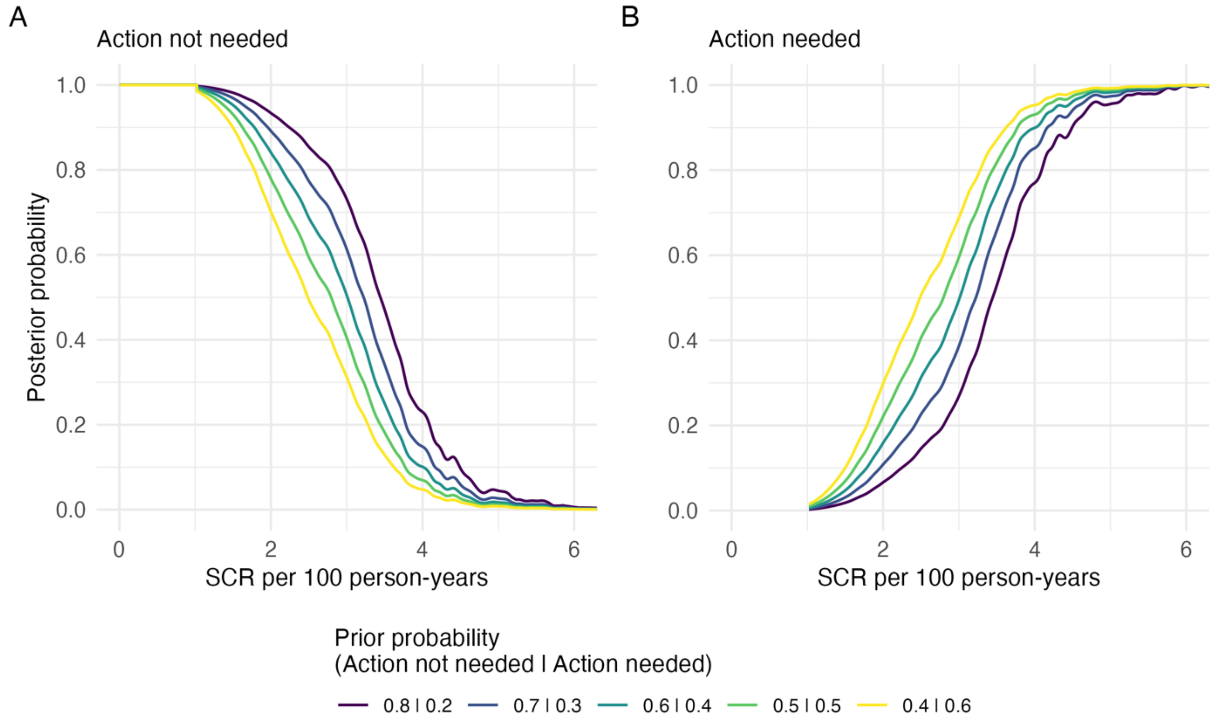

Fig S5. Comparison of Pgp3 IgG seroprevalence and seroconversion rate (SCR) estimates using different age ranges. Seroprevalence and SCR estimated at evaluation unit level in 43 surveys that used a population-based survey protocol for district or subdistrict level populations of 1–9-year-olds (n=14,620 aged 1–3 years, n=25,492 aged 1–5 years, n=43,606 aged 1–9 years). In all panels the dashed line indicates the 1:1 relationship and the solid line is a locally weighted regression fit. Blue points denote the 1–9-year-old vs 1–5-year-old comparison and red points mark the 1–3-year-old vs 1–5-year-old comparison. **A)** Seroprevalence estimated among 1–9-year-olds versus the same value estimated among 1–5-year-olds. **B)** SCR estimated among 1–9-year-olds versus the same value estimated among 1–5-year-olds. **C)** Seroprevalence estimated among 1–3-year-olds versus the same value estimated among 1–5-year-olds. **D)** SCR estimated among 1–3-year-olds versus the same value estimated among 1–5-year-olds. Although 1–5-year-old seroprevalence is systematically lower than seroprevalence among 1–9-year-olds (panel A) and higher than when estimated among 1–3-year-olds (panel C), SCR is broadly consistent when estimated using the different age ranges (panels B, D).

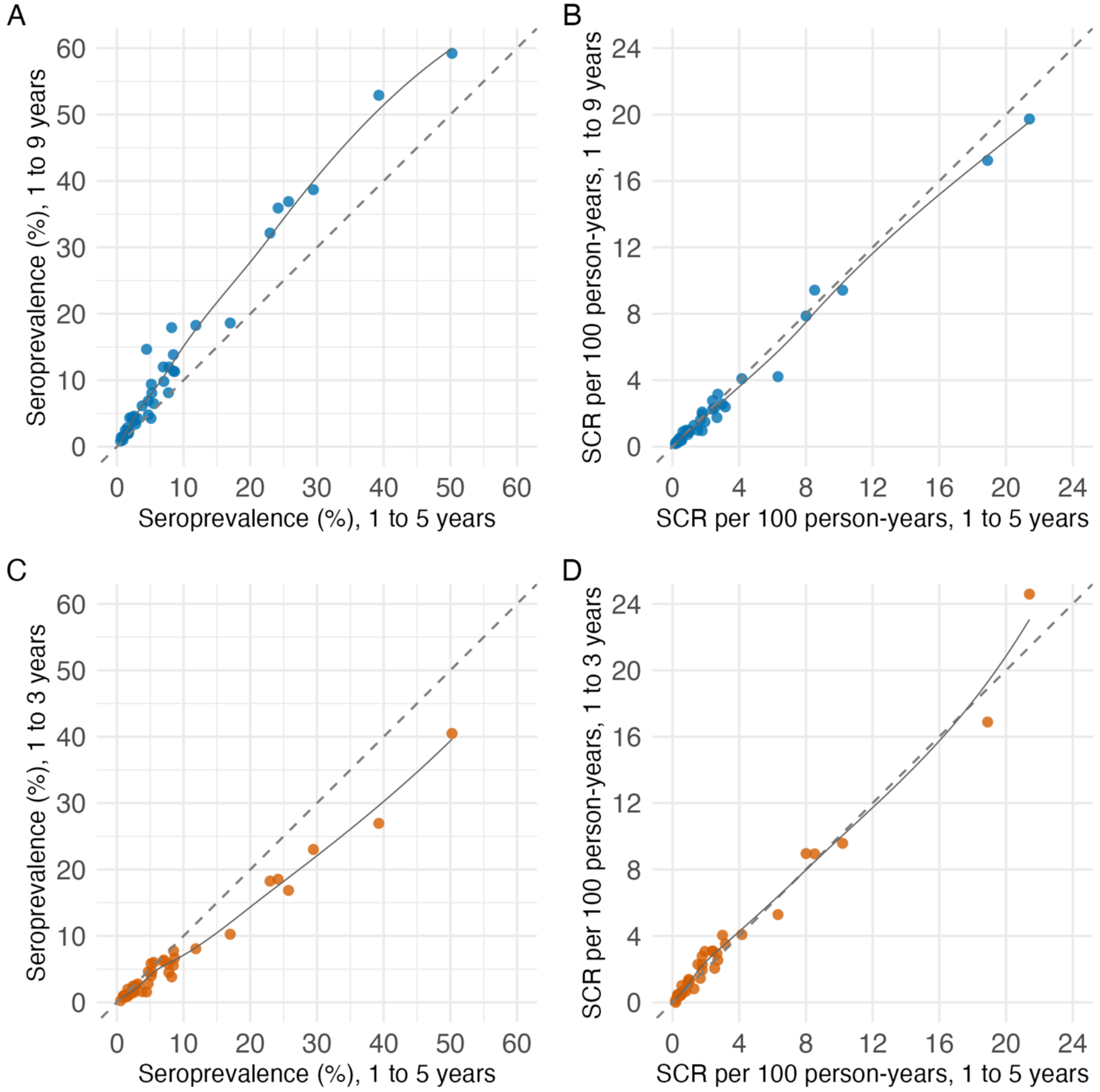

Fig S6. Comparison of (Pgp3 + Ct694) IgG responses versus Pgp3 alone for seroprevalence and seroconversion rate (SCR) estimation. (A) Seroprevalence and **(B)** SCR per 100 person-years (PY) estimated at the evaluation unit level in 36 surveys that had measured IgG responses to both Pgp3 and Ct694 antigens in 1–5-year-olds. In all panels the dashed line indicates the 1:1 relationship. The histograms summarize the differences between estimates with double versus single antigen.

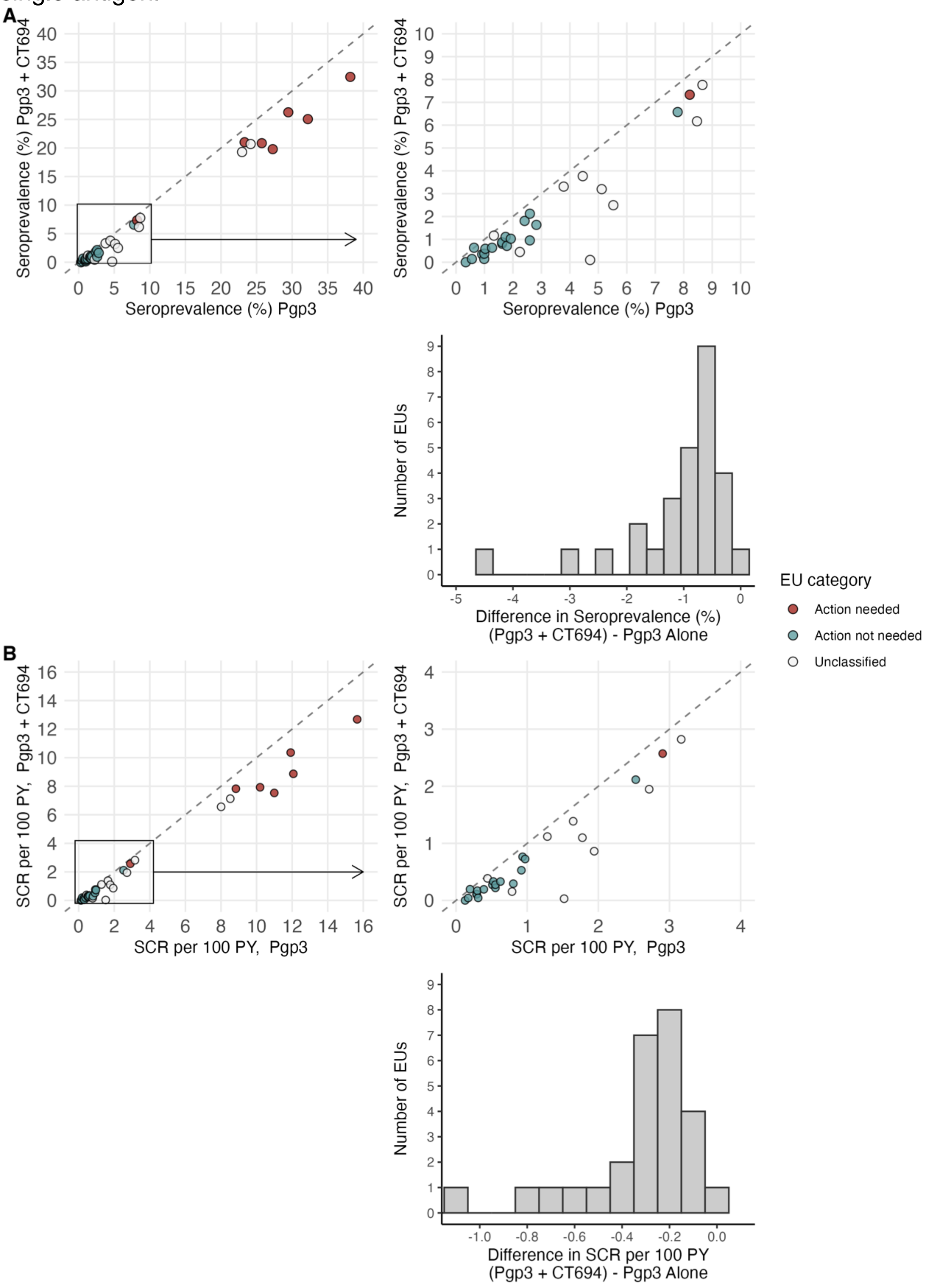

Fig S7. Comparison of the seroconversion rate (SCR) estimated using a reversible catalytic model allowing for seroreversion with a model that assumes no seroreversion. Allowing for seroreversion, here assumed to be 6 per 100 person-years based on previous estimates from longitudinal cohorts in near elimination transmission settings, scales the SCR linearly but does not change the rank order in estimates. Estimates are colored by evaluation unit (EU) trachoma programmatic decision category used in the main analyses (N=48 EUs). The dashed line indicates a 1:1 relationship and the solid line indicates a linear regression fit, with intercept 0 and slope 1.15 (adjusted R^2=1). The right panel provides a zoomed-in view of SCR estimates with values below 5 per 100 person-years.

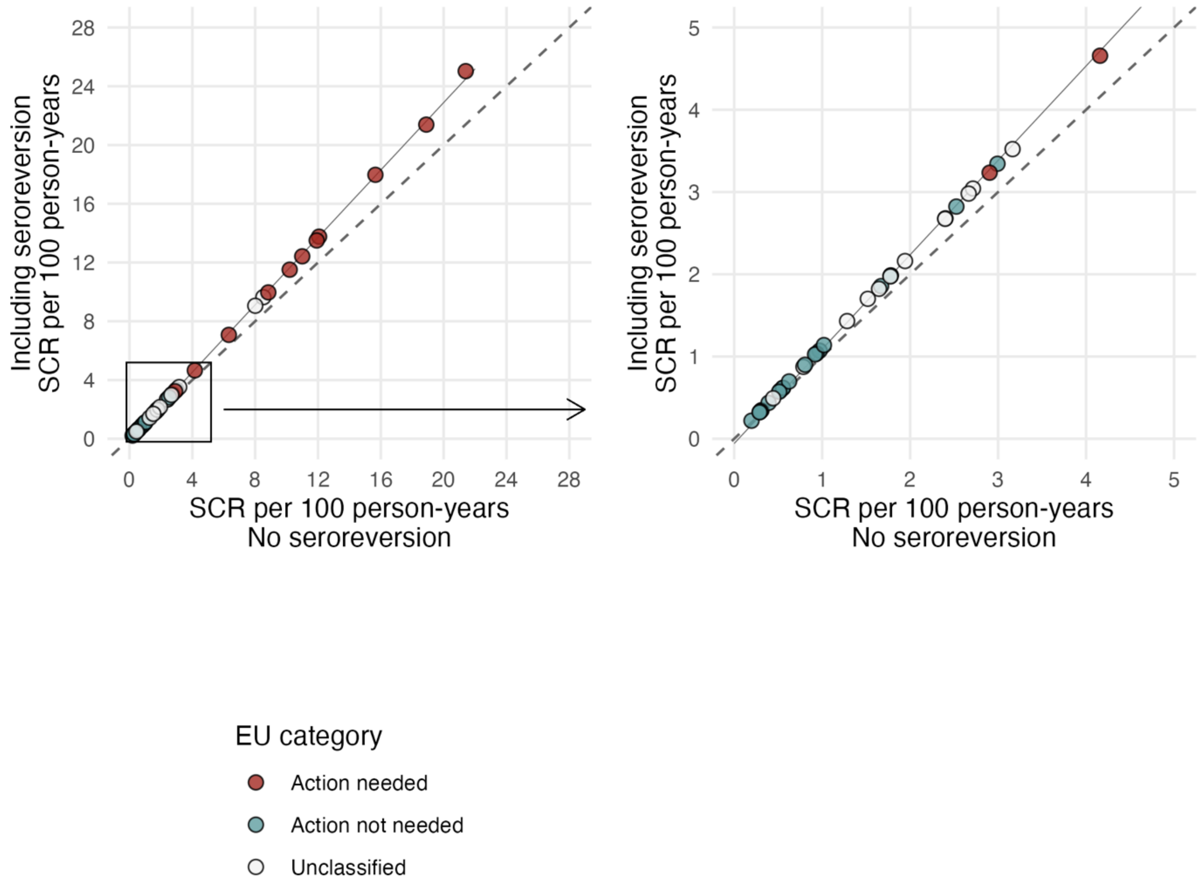

Fig S8. Comparison of Bayesian Monte Carlo Markov Chain (MCMC) estimator versus maximum likelihood with generalized linear models (GLM) estimator. Comparison of Pgp3 IgG seroprevalence (A) and seroconversion rate (B) estimated using MCMC and GLM estimators. Evaluation units (EU) are colored by transmission categories used in the main analysis (N=48 EUs). Dashed lines indicate the 1:1 relationship and right panels provide a zoomed view to estimates for populations at low levels of transmission.

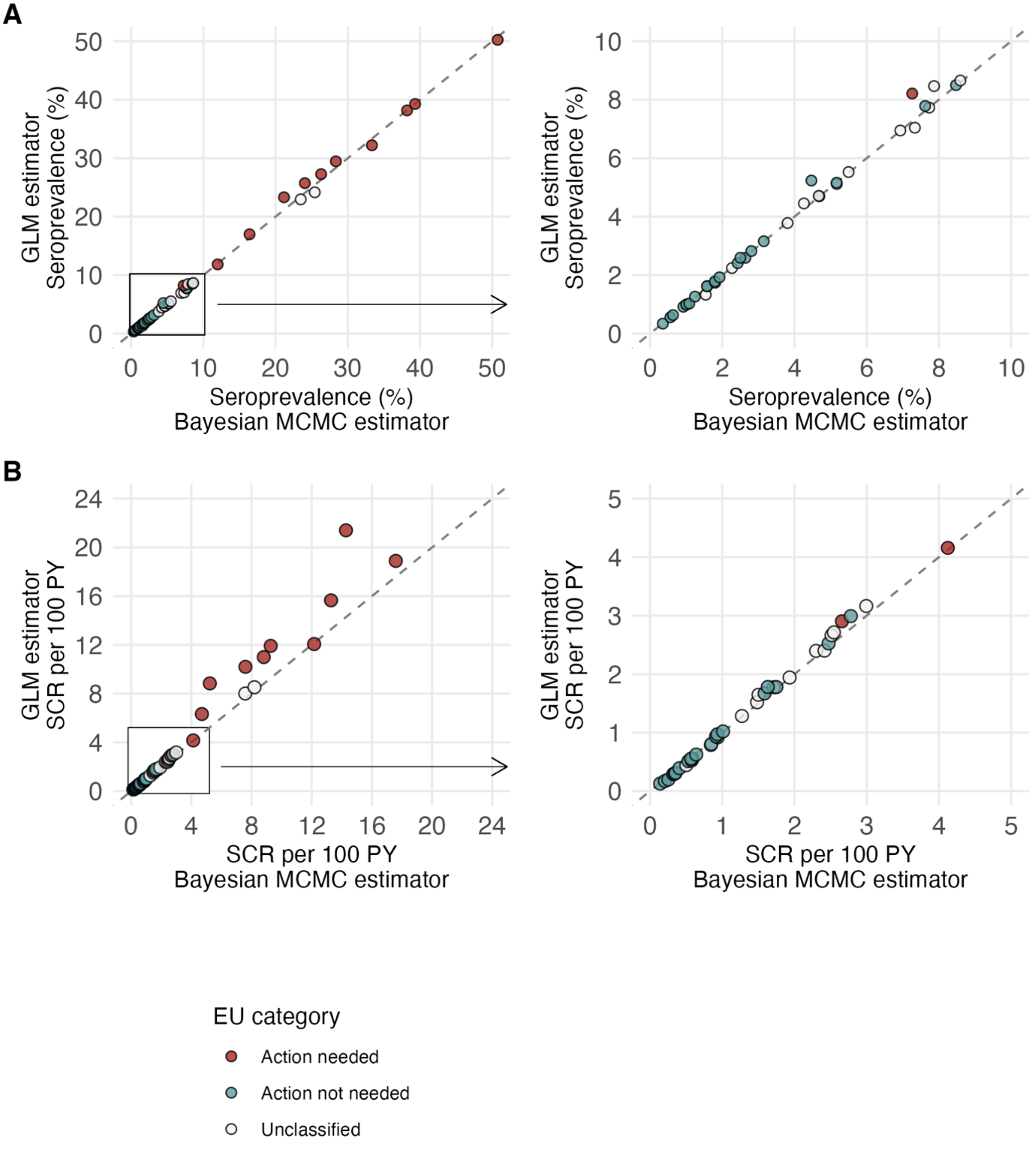

PY: person-years

Fig S9. Comparison of seroprevalence estimates with and without age standardization. Seroprevalence estimated at the evaluation unit level for three different age ranges in 39 evaluation unit surveys that used a population-based survey protocol for district or subdistrict level populations of 1–9-year-olds (n=14,620 aged 1–3 years, n=25,492 aged 1–5 years, n=43,606 aged 1–9 years). The results illustrate that age standardization (assuming uniform age distribution) has very little influence on seroprevalence estimates. **(A)** Estimates for all 43 evaluation units. The dashed line indicates the 1:1 relationship. The bounded box indicates the zoom window for panel in B. **(B)** Estimates limited to the 0–20% seroprevalence region to better visualize the data for populations at low levels of transmission.

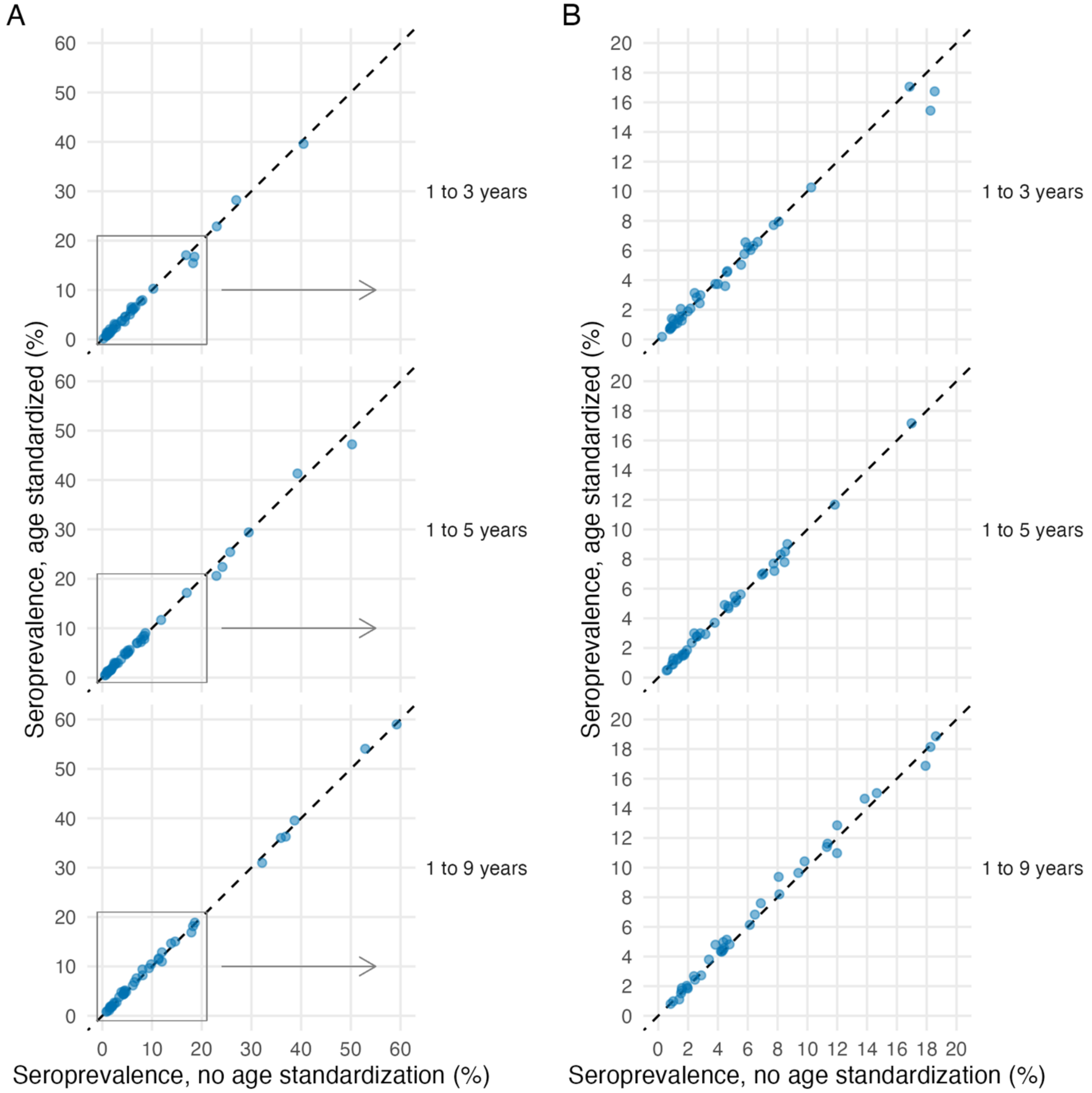

References

1. Thylefors, B., Dawson, C. R., Jones, B. R., West, S. K. & Taylor, H. R. A simple system for the assessment of trachoma and its complications. *Bull. World Health Organ.* **65**, 477–483 (1987).
2. Nash, S. D. *et al.* Prevalence of Ocular Chlamydia trachomatis Infection in Amhara Region, Ethiopia, after 8 Years of Trachoma Control Interventions. *Am. J. Trop. Med. Hyg.* **108**, 261–267 (2023).
3. Gwyn, S. *et al.* Precision of Serologic Testing from Dried Blood Spots Using a Multiplex Bead Assay. *Am. J. Trop. Med. Hyg.* **105**, 822–827 (2021).
4. Gwyn, S. *et al.* The Performance of Immunoassays to Measure Antibodies to the Chlamydia trachomatis Antigen Pgp3 in Different Epidemiological Settings for Trachoma. *Am. J. Trop. Med. Hyg.* **105**, 1362–1367 (2021).
5. Gwyn, S. *et al.* Comparison of platforms for testing antibodies to Chlamydia trachomatis antigens in the Democratic Republic of the Congo and Togo. *Sci. Rep.* **11**, 7225 (2021).
6. Gwyn, S., Mkocha, H., Randall, J. M., Kasubi, M. & Martin, D. L. Optimization of a rapid test for antibodies to the Chlamydia trachomatis antigen Pgp3. *Diagn. Microbiol. Infect. Dis.* **93**, 293–298 (2019).
7. Gwyn, S. *et al.* Comparison of Platforms for Testing Antibody Responses against the Chlamydia trachomatis Antigen Pgp3. *Am. J. Trop. Med. Hyg.* **97**, 1662–1668 (2017).
8. Gwyn, S. *et al.* Lateral flow-based antibody testing for Chlamydia trachomatis. *J. Immunol. Methods* **435**, 27–31 (2016).
9. Goodhew, E. B. *et al.* CT694 and pgp3 as Serological Tools for Monitoring Trachoma Programs. *PLoS Negl. Trop. Dis.* **6**, e1873 (2012).
10. West, S. K. *et al.* Longitudinal change in the serology of antibodies to Chlamydia trachomatis pgp3 in children residing in a trachoma area. *Sci. Rep.* **8**, 3520 (2018).
